# Is ICT Development Conducive to Reducing the Vulnerability of Low-Carbon Energy? Evidence from OECD Countries

**DOI:** 10.3390/ijerph20032444

**Published:** 2023-01-30

**Authors:** Lingling Zhou, Tao Shi, Qian Zhou

**Affiliations:** 1 Graduate School of Management, Management and Science University, University Drive, Off Persiaran Olahraga, Section 13, Shah Alam 40100, Malaysia; 2 Economics Institute, Henan Academy of Social Science, Zhengzhou 450002, China; 3 Hebi High-Quality Development Research Institution, Hebi 458030, China; 4 Economics School, Zhongnan University of Economics and Law, Wuhan 430073, China

**Keywords:** low-carbon energy, energy vulnerability, ICT, moderating effect

## Abstract

This paper constructs a low-carbon energy vulnerability system with the three dimensions of economy–society–environment, uses the entropy method to measure the low-carbon energy vulnerability index of Organization for Economic Co-operation and Development (OECD) countries from 2002 to 2018, and observes the essential characteristics. On this basis, we analyze the impact of the development of the Information Communication Technology (ICT) service industry on the vulnerability of low-carbon energy and explore the moderating effects of technological innovation and resource consumption. This paper draws the following conclusions: (1) The low-carbon energy vulnerability of OECD countries shows a gradual downward trend, showing three stages of “continuous rise—declining volatility—low-level fluctuation”. The low-carbon energy policies and implementation efforts in different countries have become the key to reducing the vulnerabilities of low-carbon energy in OECD countries. The economic and social vulnerabilities of low-carbon energy in most countries are outstanding. (2) The development of the ICT service industry benefits by reducing the vulnerability of low-carbon energy with a significant weakening effect, while high-vulnerability countries benefit even more. (3) In the weakening effect of the development of the ICT service industry on the vulnerability of low-carbon energy, technological innovation exerts an enhanced moderating effect, and resource consumption exerts a disruptive moderating effect. Technological innovation accelerates the weakening effect of the ICT service industry, and resource consumption is not conducive to the weakening effect of the ICT service industry. Based on this, we draw corresponding policy recommendations.

## 1. Introduction

Global environmental problems caused by climate change have received widespread attention from countries around the world. Excessive carbon emissions and coal energy consumption have resulted in more serious environmental pollution and damaged the healthy living environment of global residents [1]. Energy is one of the crucial driving forces leading the sustainable development of economies and societies [2]. Realizing the energy transition and promoting the harmonious coexistence of humankind and nature is an important issue faced by global governments. It is crucial to reduce energy vulnerability given the increasingly severe global climate change situation. Thus, the relative problem is as follows: how to scientifically assess energy vulnerability is important for deepening global energy governance cooperation and promoting a new round of energy reform oriented towards clean and low-carbon energy [3].

Low-carbon energy transition refers to the change from an energy supply system based on coal to an energy supply system based on low-carbon, low-emission, and high-renewable energy. The methods of low-carbon energy transition include the following: First, developing the clean use of traditional energy. Increase the clean utilization of conventional energy, such as coal and oil, to upgrade the development technology of natural gas. Improving the utilization efficiency of traditional energy is a feasible way to decrease the energy supply pressure, develop green energy technologies, save energy consumption, and enhance energy use efficiency. Second, optimizing the energy structure and promoting the diversified use of energy. Compared with coal energy, renewable energy can reduce air pollutant emissions [1]. Renewable energy has a wide distribution, great potential, and sustainable use. Renewable energy is a critical factor in solving the low-carbon emissions problem. Therefore, it has become an important choice for the energy transition. Speeding up the utilization of non-fossil energy, such as geothermal energy, wind energy, and solar energy, can accelerate the replacement of high-carbon energy with low-carbon energy and renewable energy to replace fossil energy [4]. The single energy structure dominated by coal is one of the reasons for energy vulnerability. Developing clean and renewable energy is an effective way to decrease energy vulnerability. Increasing the development and utilization of renewable energy, such as hydropower, tidal power, wind energy, solar energy, and biomass energy; strengthening infrastructure construction; and increasing the development and utilization rate of renewable energy are important ways to realize the energy transition. The third method is to improve energy storage and conversion technology and develop carbon capture, utilization, and storage. Facing the challenge of energy vulnerability, improving energy conversion and energy storage technology is necessary. Developing energy storage carriers is a key step to promoting energy transition. For example, hydrogen is an ideal energy carrier because hydrogen can refine most kinds of energy. Using fossil fuels and renewable energy to produce hydrogen energy alongside safe and low-cost energy storage will facilitate energy transportation and solve the problem of the interval allocation of energy distribution. Carbon capture, utilization, and storage (CCUS) technology is a way to convert and recycle carbon dioxide. The world produces 83 billion tons of carbon dioxide (International Energy Agency, IEA) every year. Purifying and recycling are effective ways to improve energy efficiency, ensure energy security, and reduce energy vulnerability in the future. Fourth, the opportunity of new technology information infrastructure construction to promote the digital intelligence development of energy and the entire energy industry chain could be taken in order to accelerate the synergy of multiple energy sources and the two-way interaction of energy supply and demand and to improve the efficiency of the energy system.

Throughout the last decade, scholars have analyzed the research on the vulnerability of low-carbon energy from multiple perspectives, such as energy transition, energy structure, and energy technology. A few scholars have paid attention to low-carbon energy vulnerability assessment. The analysis of the relationship between intelligent transition and the vulnerability of low-carbon energy, especially the empirical basis based on the sample of OECD countries in the energy transition pioneer area, provides space for the study of this article. Although most OECD countries have relatively complete energy supply systems, market supply and demand trends are still affected by COVID-19, and a low-carbon energy transition is imperative. To this end, this paper will analyze the impact of ICT technology on the vulnerability of low-carbon energy and its mechanism to provide empirical evidence for the global low-carbon energy transition.

The rest of this article is as follows: the second part is a literature review and the third part evaluates and analyzes low-carbon energy vulnerability indicators. A low-carbon energy vulnerability evaluation indicator system based on OECD countries is constructed and analyzed using three-dimensional graphs. The fourth part is the analysis of the impact of the ICT service industry development on the vulnerability of low-carbon energy, exploring the benchmark model and the heterogeneity analysis of the effects of ICT on the vulnerability of low-carbon energy; the fifth part is the analysis of the impact mechanism of the development of the ICT service industry on the vulnerability of low-carbon energy, with the moderating effects of technological innovation and resource consumption; and the sixth part comprises the conclusion and policy implications.

## 2. Literature Review

### 2.1. Low-Carbon Energy Vulnerability

Low-carbon energy refers to a type of energy that replaces high-carbon energy, mainly involving renewable energy, such as electric energy, tidal energy, geothermal energy, and other zero-carbon or very low-emission energy sources [5,6]. At present, scholars have not yet formed a consensus on the definition of low-carbon energy vulnerability, and energy vulnerability is being increasingly discussed. Gnansounou [7] believes that energy vulnerability refers to the ability of the energy system to respond to specific emergencies and adverse effects. Similarly, Gatto and Busato [3] defined energy vulnerability as the degree of risk that the energy system is more susceptible to social changes and significant events and that the economy, society, and the environment are more likely to fall into trouble. G. Wu [8] defines energy vulnerability as the uncertainty and insecurity of the energy supply. Genave et al. [2] define energy vulnerability as the sensitivity degree of the energy system to the threat of potential external adverse events and the regular operation of the available technologies for production, transportation, and distribution within the energy system. That is, the stability of energy when internal and external environmental changes impact it. A few scholars have discussed the concept of low-carbon energy vulnerability. For example, B. Wang et al. [4] defined the vulnerability of renewable energy as the exposure, sensitivity, and adaptability of the renewable energy system to climate change. Here, the degree of exposure refers to the impact of climate change caused by human factors on the ecological environment, involving five parts: extreme weather events, land use status, population exposure, economic exposure, and energy structure exposure; sensitivity refers to the extent to which a region dependent on renewable energy; and adaptive capacity refers to the potential of using renewable energy systems to mitigate the effects of climate change.

It can be seen that the above-mentioned scholars’ definition of energy vulnerability reflects an important feature, that is, the ability to deal with the impact of uncertainty. Under the framework of the Paris Agreement, responding to global climate change, global low-carbon energy has become the basic consensus of all countries in the world. At the same time, under the interference of major uncertain factors, such as COVID-19, global economic development has increased in instability, thereby increasing the security of energy supply affected by geopolitics, transportation, and price risks; hence, increasing the vulnerability of low-carbon energy. For this reason, based on the research results of scholars and the global consensus, we define the vulnerability of low-carbon energy as: renewable energy, such as solar energy, wave energy, biomass energy, wind energy, or very low-carbon emission energy, to respond to the impact of uncertain events, such as climate change, political economy, etc. [5,6].

### 2.2. ICT Service Industry Development and Low-Carbon Energy Vulnerability

In the 21st century, global environmental issues have become important challenges for the development of human society. Reducing carbon dioxide emissions and promoting energy transition are the main ways of improving environmental problems. The European Union’s (EU) newly established competitive and innovative plan, Smart Energy Europe; ICT; and innovation policy are listed as three pillars. With the advent of the era of intelligent energy, information and communications technology can improve energy production and use via the following aspects: through big data analysis and computer cloud computing, real-time energy consumption can be collected in real-time and automation, such as demand response and sensor technology, can be applied. The operating system completes the required energy output forecast, realizes energy forecast automation, and distributes energy through the distributed power generation and smart grids, which reduces energy storage costs and avoids energy waste simultaneously, while improving energy production efficiency through early warning management, predictive maintenance, machine learning, and other technologies. At the same time, energy management and energy consumption systems are combined to form an intelligent energy urban public service system. By establishing an information exchange platform, remote access to the user interface to collect energy production and real-time information on distribution and trade improves energy production and consumption efficiency [9].

The increase in technology means can realize the intelligent management and consumption of energy to a certain extent and reduce the vulnerability of energy. Intelligent management and the use of energy have become the trends of future energy development. ICT services can provide technical solutions for green and sustainable development [10]. For example, digital operations and regional power integration can improve industrial energy efficiency [11], achieving energy saving, emission decrease, and environmental protection effects. The development of ICT service infrastructure is an important factor in future smart grid construction [12]. In practice, the Italian government has built an energy network plan based on information and communication technology development and proposed an energy network plan that integrates smart energy storage and on-demand power dispatch [13]. At the same time, based on seven projects in the United States (US), Germany, and Italy, the impact of the country’s mobile communication technology on renewable energy power generation shows that renewable energy will be increased by 0.2% in the long term and 1.1% in the short-term, with a 1% increase in ICT. This indicates that ICT services can reduce energy vulnerability by promoting renewable energy development [14]. More scholars have discussed the emission reduction effects of ICT and believe that the green use of information and communication technologies can decrease carbon dioxide emissions and promote energy efficiency to a certain extent [15,16,17,18], especially in the “Belt and Road” countries. More traditional industries have benefited from the development of ICT, chosen high-efficiency energy and low-carbon development, and reduced fossil fuel consumption through the development of green energy projects to reduce the level of carbon dioxide emissions [19].

However, ICT investment can contribute to the reduction of energy consumption to a certain extent [20], and ICT services in different countries will have different effects on power consumption and energy efficiency [21]. Digitization promotes energy efficiency and also causes the rebound effect to increase energy consumption [22]. Lange, Pohl, and Santarius [23] studied the energy efficiency of ICT in the building sector in 23 European countries. By implementing different ICT solutions, 50% of the countries have achieved more than 20% of their energy-saving targets. The reason for countries failing to achieve this target may be that the popularization of electronic equipment has increased power consumption. It can be seen that whether the development of the ICT service industry is conducive to the vulnerability of low-carbon energy still requires more empirical evidence. Therefore, based on the research results of scholars, this article proposes Hypothesis 1:

**Hypothesis 1 (H1).**  *The development of the ICT service industry has a weakening effect on the vulnerability of low-carbon energy.*

### 2.3. Resource Consumption and Low-Carbon Energy Vulnerability

Electricity is the main form of low-carbon energy; however, the decarbonization of electricity depends on low-cost wind, solar, and hydropower technologies and other dispatchable renewable energy sources [24]. Replacing oil and natural gas with renewable energy sources will promote the realization of resource sustainability [25], as will optimizing the resource consumption structure and improving the efficient use of low-carbon energy, such as geothermal energy, hydropower, wind energy, etc., which relies on nature to decrease the traditional energy use represented by fossil energy and strengthens the promotion and application of clean energy. This can improve low-carbon energy conversion and utilization efficiency [26], as would build a distributed energy utilization system dominated by diversified low-carbon energy, such as hydrogen fuel power generation and solar photovoltaic power generation [27]. This is an effective and feasible way to decrease the vulnerability of low-carbon energy through the central energy supply system to meet the different needs of energy users to maximize the resource consumption rate [28]. Furthermore, Z. Wang et al. [29] believe that promoting low-carbon buildings and implementing building renovations reduces the use of fossil energy in building materials, equipment, construction, etc.

Meanwhile, reducing energy consumption and paying attention to low-carbon environmental protection are important measures to alleviate energy pressure, save natural resources and impel the low-carbon transformation of cities. Moreover, promoting low-carbon transportation is a key method of reducing resource consumption and traditional fossil energy-based high-energy transportation methods [30], as is advocating for low-energy, low-emission, and energy-efficient transportation methods. For example, it is helpful to reduce the cost of carbon energy vulnerability by using hydrogen and ammonia zero-carbon synthetic materials in transportation [31], and implementing systematic low-carbon equipment, increasing energy use and resource utilization efficiency, and changing the structure of resource consumption. The environmental analysis results of Azam, Rafiq, Shafique, and Yuan [32] show that renewable energy and ICT trade can help eliminate carbon dioxide emissions, especially in high-income countries [33], while the resource consumption intensity of middle- and high-income countries will be significantly lower than that of low-income countries. Resource consumption plays a moderating role in the weakening effect of the development of the ICT service industry on the vulnerability of low-carbon energy. For this reason, based on the research results of scholars, this article proposes Hypothesis 2:

**Hypothesis 2 (H2).**  *Resource consumption plays a positive regulatory role in the weakening effect of the development of the ICT service industry on the vulnerability of low-carbon energy.*

### 2.4. Technological Innovation Resource Consumption and Low-Carbon Energy Vulnerability

The development of technological innovation is an active choice for humankind in the face of climate change [34]. Technological innovation has a particular cumulative effect on reducing carbon emissions [35]. It is an effective way to solve the problems of improving the environment [36] and the low-carbon energy vulnerability in the future. Renewable energy has the characteristics of cleanliness and sustainability. Technological innovation is favorable to facilitating renewable energy development [37], and continuously reducing the cost of renewable energy [38], especially promoting the implementation of renewable energy [39] to reduce dependence on fossil fuels while reducing nitrogen oxides (NOx) [40] and CO_2_ emissions [41,42]. The development of renewable energy technologies, such as wave energy, geothermal energy, and wind energy, to build a low-carbon technology innovation systems is conducive to reducing the vulnerability of low-carbon energy [43] and achieving energy security and environmental protection [44].

Meanwhile, technological innovation stimulates renewable energy production and consumption, transforms and develops energy consumption structure [45], improves energy efficiency [42,46], and changes regional energy intensity. At present, industrialized countries are striving to take the path of power generation and decarbonization and develop renewable energy technologies to meet the increase in power demand. Promote the research of new energy technologies, such as carbon capture and storage technologies and clean energy technologies [47]. Accelerating the formation of a cluster of green energy industries driven by technological innovation [48] will help reduce energy intensity [49,50] and promote regional energy conservation and carbon emission reduction [51], achieving the goal of reducing the vulnerability of low-carbon energy [52]. In addition, as pointed out above, ICT technology will increase power consumption and the burden of low-carbon energy. We also see the use of technological innovations, such as computer cloud services, to control the scale of energy consumption, reduce the carbon footprint, and reduce environmental harm. It is vital to reduce the carbon footprint [53]. Therefore, the degree of technological innovation restricts the development of the ICT service industry to a certain extent regarding the vulnerability of low-carbon energy. For this reason, based on the research results of scholars, this article proposes Hypothesis 3:

**Hypothesis 3 (H3).**  *Technological innovation plays a negative regulatory role in the weakening effect of the development of the ICT service industry on the vulnerability of low-carbon energy.*

### 2.5. Summary

**Figure 1:**
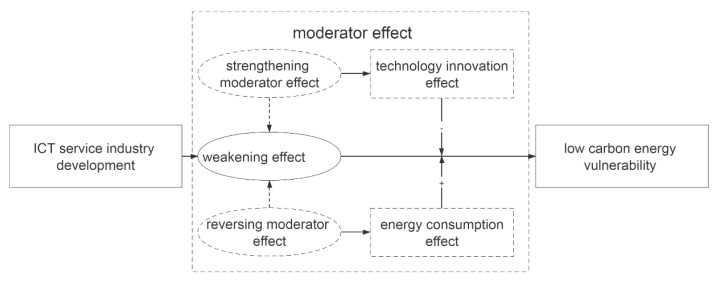
The theoretical framework.

In summary, scholars have produced extensive studies on the vulnerability of ICT development of low-carbon energy. Researchers generally believe that ICT development is conducive to improving energy use, reducing energy intensity, saving resource consumption, and reducing the vulnerability of low-carbon energy. In particular, it has obvious emission reduction effects and can reduce the adverse impact on the environment [17], which provides a solid foundation for this article’s research. However, scholars have yet to directly answer the direct impact of the development of the ICT service industry on the vulnerability of low-carbon energy. More studies have analyzed the impact of technological innovation and changes in energy consumption intensity on the vulnerability of low-carbon energy from the perspectives of technological innovation and resource consumption. The impact has not been clearly defined as the role of technological innovation and changes in resource consumption in the process of the direct impact of the development of the ICT service industry on the vulnerability of low-carbon energy. Based on the scholars’ research results, this paper constructs the impact of ICT service industry development on low-carbon energy vulnerability and its mechanisms (as shown in Figure 1). This research posits that the development of the ICT service industry has a weakening effect on low-carbon energy vulnerability. Technological innovation and resource consumption play a moderating effect. At the same time, this article will: (1) build a low-carbon energy vulnerability assessment index system and use the entropy method to evaluate the low-carbon energy of 24 OECD countries from 2002 to 2018, as well as use the Kernel nuclear density method to analyze the dynamic evolution of the low-carbon energy vulnerability level of OECD countries; (2) analyze the impact and heterogeneity of the development of the ICT service industry on the vulnerability of low-carbon energy and verify the weakening effect of the former on the latter; and (3) Analyze the adjustment mechanism of technological innovation and resource consumption in the weakening effect of the development of the ICT service industry on the vulnerability of low-carbon energy.

## 3. Evaluation and Analysis of Low-Carbon Energy Vulnerability Index

### 3.1. Low-Carbon Energy Vulnerability Assessment Design

#### 3.1.1. Index System

Ensuring the availability of energy for all is the goal of the United Nations (UN) for sustainable development and a key link in solving energy poverty and reducing energy vulnerabilities. The scientific assessment of current energy vulnerability is an effective response to emergencies, reducing the adverse impact of fluctuations in energy market supply and demand on social development, and an important way to deal with energy vulnerability. Current scholars have conducted much research on energy vulnerability assessment. In a comprehensive index of oil vulnerability, Gupta [54] uses oil consumption per unit of gross domestic product (GDP) and oil share in total energy supply, etc. Wu [8] constructed energy supply vulnerability indicators from the perspectives of energy import diversification, per capita energy consumption, energy self-sufficiency rate, energy intensity, and energy diversification. Gatto and Busato [3], from the perspectives of economy, society, environment, and governance, constructed a global energy vulnerability index (GEVI), including energy consumption, renewable energy consumption, and energy imports. Genave [55] constructed an energy vulnerability assessment framework from five dimensions: energy access, energy import dependence, GDP energy intensity, energy structure diversification, and energy bills. Gupta [54] constructed an index of energy vulnerability through the three dimensions of economy, society, and environment. Economic vulnerability includes energy import dependence, energy structure diversification, energy system conversion efficiency, GDP energy intensity, and other indicators.

As pointed out above, compared with energy vulnerability, low-carbon energy vulnerability pays more attention to the low-carbon nature of energy, especially the cleanness and sustainability of energy. To this end, based on the achievements of the above scholars on energy vulnerability, this article will highlight low-carbon energy vulnerability. Construct a low-carbon energy vulnerability evaluation index system containing 14 indicators from the three dimensions of society, economy, and environment, and evaluate the low-carbon energy vulnerability of OECD countries, as shown in Table 1.

#### 3.1.2. Evaluation Method Design

At present, scholars use methods such as principal component analysis [7], the ecological macroeconomics model, and the multi-layer benefit-of-the-doubt model [54]. The assessment of energy vulnerability has intense subjectivity or measurement characteristics that do not meet exponential indicators. Compared with other methods, the entropy method can effectively avoid the subjectivity of index weighting [56] and comprehensively evaluate the indicators objectively. Table 2 shows a comparative methodology and analysis for other reported works.

Following previous research [56], we use the entropy method to calculate the low-carbon energy vulnerability index, including:

(1) $$\left\{ \begin{array}{l} x_{i}^{\prime }=\frac{x_{i}-\min\{x_{1},\ldots ,x_{n}\}}{\max\{x_{1},\ldots ,x_{n}\}-\min\{x_{1},\ldots ,x_{n}\}}\\ p_{i}=(1+x_{i}^{\prime })/\sum_{i=1}^{n}\left(1+x_{i}^{\prime }\right)\\ e_{j}=-k\sum_{i=1}^{n}p_{i}\times \ln(p_{i}),k=1/\ln(n) \end{array}\right.$$

In Formula (1), *x_i_* is the indicator of sample *i* in Table 1, where i∈[1,…,n]. $x_{i}^{\prime }$ is the positive normalization value of *x_i_*. Compared to this, the negative normalization form only needs to change the numerator of $x_{i}^{\prime }$ into $ax\left\{x_{1},\ldots ,x_{n},\right\}-x_{i}$. *p_i_* is the sample index weight, *e_j_* is the information entropy of the *j*-th index, *k* is the adjusted parameter, and *n* is the sample size. Therefore, weights of different samples *w_j_* of dimension *j*:

(2) $$w_{j}=d_{j}/\sum_{j=1}^{m}d_{j},d_{j}=1-e_{j}$$

In Formula (2), $w_{j}$ is the weights of different samples and *d_j_* is the utility value of the *j*-th index. We obtain the low carbon energy vulnerability index $tindex_{i}$:

(3) $$tindex_{i}=\sum_{j=1}^{m}w_{j}\times x_{i}^{\prime }$$

#### 3.1.3. Data Source and Description

The above data are from the World Bank and Economy Prediction System (EPS) databases without special instructions. Subject to data availability constraints, the OECD countries selected in this article are 24, including Israel, Canada, Hungary, South Africa, Colombia, Turkey, Mexico, Austria, Greece, Germany, Italy, Norway, Czech Republic, Belgium, France, Poland, Sweden, Switzerland, the United States, Finland, the United Kingdom, the Netherlands, Portugal, and Spain. The sample time range is from 2002 to 2018, and some missing values are calculated using 5-year moving smoothing calculations.

### 3.2. Low-Carbon Energy Vulnerability Analysis of OECD Countries

#### 3.2.1. Analysis of the Trend Characteristics of the Vulnerabilities of Low-Carbon Energy in OECD Countries

**Figure 2:**
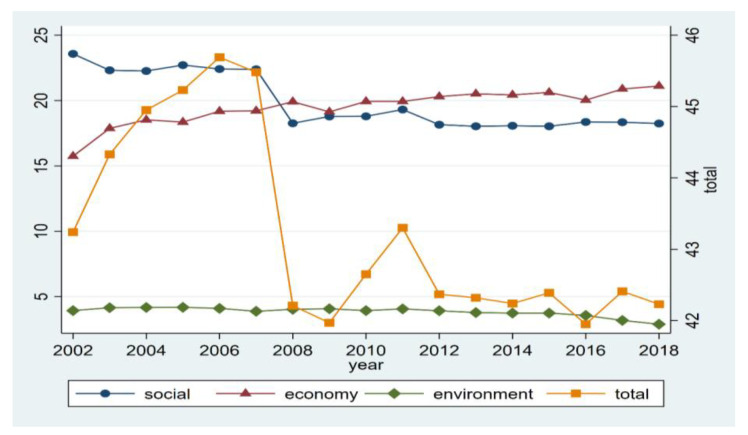
Trends in the vulnerabilities of low-carbon energy in OECD countries from 2002 to 2018. Note: The magnitude of the left vertical axis refers to the social vulnerability, economic vulnerability, and environmental vulnerability. The axis unit is 100%.

Figure 2 shows the trend of low-carbon energy vulnerabilities in OECD countries from 2002 to 2018. From this figure, we can see that, overall, the low-carbon energy vulnerability of OECD countries from 2002 to 2018 showed a fluctuating downward trend, which can be precisely divided into three stages. The first stage is from 2002 to 2007, the low-carbon energy vulnerability of OECD countries fluctuated upward, and the overall low-carbon energy vulnerability level was maintained at a high level, even reaching 45.69% in 2006.

In the second stage, from 2008 to 2011, the vulnerabilities of low-carbon energy in OECD countries decreased significantly compared with the previous stage. The overall fluctuations showed a slight upward trend. During this phase, the Obama administration of the United States proposed Corporate Average Fuel Economy (CAFE) standards for clean power plans and fuel economy. Meanwhile, the European Union formulated unified low-carbon energy action plans, such as “Climate Action and Renewable Energy Package Plan” (2008) and “National Renewable Energy Action Plan” (2009), “European Union 2020 Strategy: A Strategy for the Development of Competitive, Sustainable and Safe Energy” (2010), “European Strategic Energy Technology Plan” (2010) and “Roadmap for a Powerful Low-Carbon Economy Towards a More Competitive Strategy in 2050” (2011), which effectively reduced the vulnerability of low-carbon energy. During this period, the OECD countries, including the EU and the United States, formulated policies that focused on carbon emission reduction targets and focused more on the path and strategy of low-carbon strategies, which effectively reduced the vulnerability of OECD countries to low-carbon energy to a certain extent. However, it did not fundamentally grasp the endogenous motivation to reduce the vulnerability of low-carbon energy, which led to a slight rebound in the vulnerability of low-carbon energy around 2011.

The third stage is from 2012 to 2018, when OECD countries’ low-carbon energy vulnerability fluctuated. In this stage, the low-carbon policies of OECD countries were more focused on low-carbon energy technology research and development, such as low-carbon technology innovation, including wind energy, solar energy, biomass, smart grid, electric vehicles, nuclear energy, carbon capture, and storage. The rapid industrialization of renewable energy and new energy utilization technologies accelerated. Especially around 2011, Spain, Denmark, Italy, France, the United Kingdom, and Germany formulated the “National Renewable Energy Action Plan” and “Renewable Energy Roadmap”, which cleared the goal of renewable energy development and greatly promoted the reduction of the vulnerabilities of low-carbon energy in OECD countries. However, the Trump administration of the United States pursued fossil energy and withdrew from the Paris Agreement in 2017 to pursue US energy independence. To a certain extent, the vulnerability of OECD countries to low-carbon energy increased slightly after 2016.

#### 3.2.2. Analysis of the Dimensional Structure Characteristics of the Vulnerabilities of Low-Carbon Energy in OECD Countries

From the perspective of the structure of energy vulnerability in OECD countries, in 2018, low-carbon energy had the highest economic vulnerability, followed by social vulnerability and environmental vulnerability. Specifically, because of the advancement of low-carbon energy plans by most countries in the EU as the main representative since 2005 and maintaining good policy continuity, the low-carbon energy environmental vulnerability of OECD countries has always been at a relatively low level. It dropped from 3.93 in 2002 to 2.89 in 2018, with an average annual decline of 1.91%, showing a clear downward trend, becoming the main driving force for weakening the energy vulnerability of OECD countries. At the same time, the social vulnerability of low-carbon energy has also continued to decline, from 23.54 in 2002 to 18.24 in 2018. For the first time in 2008, it was lower than the economic vulnerability of low-carbon energy. However, the economic vulnerability of low-carbon energy continued to rise, increasing from 15.74 in 2002 to 21.11 in 2018. As pointed out above, under the direct impact of the US subprime mortgage crisis in 2008 and the European debt crisis in 2011, in order to stabilize economic growth, some countries relaxed their requirements for low-carbon energy development. Thus, the low-carbon energy vulnerability of OECD countries maintained a relatively low level of growth. Moreover, the introduction of the Fossil Energy Program in 2016 in the United States contributed to the vulnerability of low-carbon energy in OECD countries to a certain extent.

#### 3.2.3. Analysis of the Structural Characteristics of the Vulnerabilities of Low-Carbon Energy in OECD Countries

**Figure 3:**
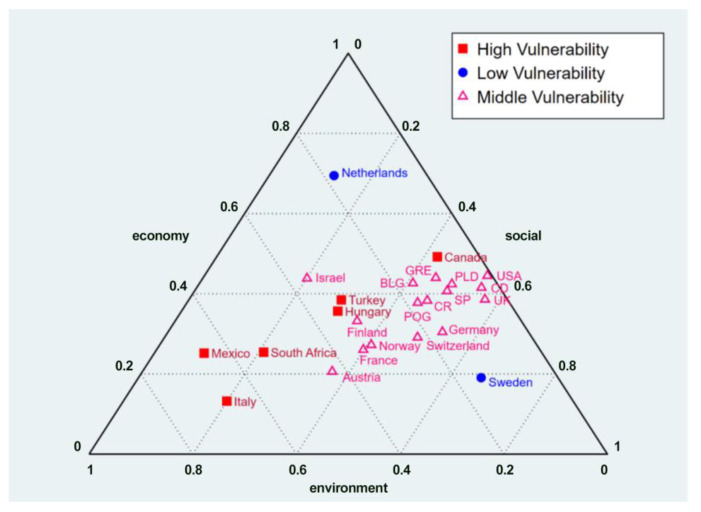
Structural analysis of low-carbon energy vulnerability in OECD countries in 2018. Note: BLG, CO, CR, GRE, PLD, POG, and SP are the abbreviations of Belgium, Columbia, Czech Republic, Germany, Poland, Portugal, and Spain, respectively.

Further, we analyze the structural issues of low-carbon energy vulnerability in different countries or regions, as shown in Figure 3. Based on the average (0.4223) and standard deviation (0.1359) of low-carbon vulnerability value in 2018, three levels are constructed: low-, middle- and high-level low-carbon vulnerability, for which the corresponding low-carbon vulnerability ranges are lower than 0.2864 and 0.2864–0.4223 and higher than 0.4223, respectively. From Figure 3, we can see that there are few low-level countries, and these are mainly located in Northern Europe, including Sweden and the Netherlands. Of these, Sweden’s renewable energy relies on nuclear power and hydropower, their low-carbon energy is too concentrated, their per capita renewable energy is low, and their social vulnerability of low-carbon energy vulnerability is relatively high. At the same time, Netherlands’ energy structure is relatively simple, mainly oil, natural gas, and coal. Renewable energy, such as wind, solar, and biomass power generation, is relatively small, and the renewable energy rate per unit of GDP is relatively low. The economic vulnerability of low-carbon energy is relatively high. Second, there are many low- and middle-level countries, which are widely distributed. These countries have high economic and social vulnerabilities, especially relatively high social vulnerabilities, which have become the key to restricting low-carbon energy vulnerabilities. Third, there are a relatively high number of high-level countries. These are mainly OECD countries with relatively low economic levels. Similar to low- and middle-level carbon-vulnerable countries, economic and social vulnerability are also high. However, the economic vulnerability of high-level low-carbon vulnerability is significantly higher than that of others and is much higher than that of low-carbon social vulnerability, especially high-carbon emission energy, such as oil and coal, which are still the primary economic energy sources in OECD countries, such as South Africa, Mexico, and Turkey. The proportion of low-carbon energy in GDP units is relatively low. An increasing economic vulnerability has become an essential constraint on the vulnerability of low-carbon energy in such countries.

## 4. Analysis of the Impact of the Development of ICT Service Industry on the Vulnerability of Low-Carbon Energy

### 4.1. Research Design

A benchmark model of the impact of ICT service industry development on low-carbon energy vulnerability is constructed:

(4) $$tindex_{it}=c_{0}+\alpha \times ict\_exp_{it}+\beta \times \sum X_{it}+\mu_{it}+\varepsilon_{it}+\upsilon_{t}$$

In Formula (4), *ict_exp_it_* is the development level of the ICT service industry in region *i* and year *t*; *tindex_it_* is low-carbon energy vulnerability; *X_it_* represents control variables; *μ_it_* and *ε_it_* are time effect and individual effect, respectively; $\upsilon_{i}\;$ is a random error term; $c_{0}$ is the constant term; and *α* and *β* are coefficients. Furthermore, this paper will use the Sivqr (Smoothed IV quantile regression) model based on Equation (4) to investigate the heterogeneity of the reduction effect of the ICT service industry development level on the low-carbon energy vulnerability [58].

### 4.2. Variable Selection and Data Source Description

#### 4.2.1. Variable Selection

Dependent variable: low-carbon energy vulnerability (*tindex*) is derived from the above comprehensive low-carbon energy vulnerability index.

Core variables: the level of ICT service industry development (*ict_exp*) is represented by the total export of ICT services.

Instrumental variables: the coverage of secure internet servers (*intser*) is the infrastructure for the development of information and communication technology. The more comprehensive the coverage, the higher the level of informatization and digitization in the region, and the more it can promote the improvement of the development level of the regional ICT service industry. Vulnerability is not directly related; the mobile phone usage rate (*iv_moble*) reflects the degree of development of the regional ICT service industry from one side. The higher the mobile phone usage rate, the higher the development level of the ICT service industry, which is not directly related to the vulnerability of low-carbon energy. For this reason, the coverage of secure internet servers and mobile phone usage can be used as instrumental variables for the level of ICT services. In order to test the rationality of the instrumental variables, this article will use the weak instrumental variable test and the identified test to identify the rationality of the relevant instrumental variables. Of these, the horizontal coverage rate of secure internet servers is represented by the number of secure internet servers per million people. The mobile phone usage rate is represented by the number of phones rented by the mobile cellular wireless communication system per 100 people.

Moderating variables: technological innovation level (*r_rd2*), considering that enterprise innovation can better reflect the regional technological innovation than resident innovation. It was chosen to be represented by the number of non-resident patent grants [50]. Resource consumption level (*re_gdp*) is expressed by energy consumption efficiency [59].

Control variables: in order to minimize the endogenous problems caused by the omitted variables, this section controls the relevant variables as much as possible under the premise of data availability. Industrial development level (*p_indty*), expressed by the proportion of industrial increase in GDP [60]; economic development level (*agdp*), expressed by GDP per capita [61]; population aging (*r_older*), expressed by the proportion of the population aged 65 and over in the total population [62]; population density (*r_density*), expressed by the number of people per kilometer of land area [63]; fiscal pressure (*e_gdp*), expressed by the proportion of fiscal revenue and expenditure gap in GDP [64]; and credit scale (*pcrd*), expressed by the domestic credit provided by the financial sector in GDP [65].

#### 4.2.2. Data Source Description

Unless otherwise specified, the above data are from World Bank, Economy Prediction System (EPS) database and International Monetary Fund (IMF) database. Considering the data availability, the sample time range is from 2002 to 2018, and finally, the panel data of 24 OECD countries are constructed. Some variables’ default values are calculated using a 5-year moving smoothing calculation. In order to reduce the influence of variance, this paper performs logarithmic processing on all variables, and the descriptive statistics of related variables are shown in Table 3.

### 4.3. The Relationship between the ICT Industry Development and Energy Vulnerability

Table 4 shows the estimation results of the benchmark model. In column (1), the coefficient of ln*ict_exp* is significantly negative at the 1% confidence level, indicating that the development of ICT technology industry servicing is conducive to reducing low-carbon energy vulnerability. After adding the control variables, the estimated coefficients of OLS, 2SLS, and IV-GMM are significantly negative at the 1% confidence level, which are basically the same as the results before the control variables were added. Still, the coefficients are all greater than −0.0845, indicating that the endogeneity problem has weakened the impact of the development of the ICT service industry on low-carbon energy vulnerability. At the same time, it further confirms that the improvement of the ICT service industry in OECD countries can reduce low-carbon energy vulnerability. Hypothesis 1 is established. In high-income and middle-income countries, the use of information and communication technologies in transportation and industrial systems can improve low-carbon energy efficiency and reduce carbon dioxide emissions and is an effective way to develop clean energy [33].

### 4.4. Heterogeneous Analysis of the Impact of ICT Service Industry Development on the Vulnerability of Low-Carbon Energy

The above proves that the development of the ICT service industry has the effect of reducing low-carbon energy vulnerability. However, it does not analyze the weakening effect of different degrees of low-carbon energy vulnerability. Countries with different low-carbon energy vulnerabilities have different effects. For this reason, this article chooses the five representative points of 10%, 20%, 50%, 75%, and 90% to classify OECD countries’ low-carbon energy vulnerability levels, based on model (1) using the Sivqr mothed to analyze the effect of ICT service industry to low-carbon energy vulnerability. It also characterizes the ICT service industry and the marginal contribution rate of different factors to different low-carbon energy vulnerabilities. The estimated results are shown in Table 5.

In Table 5, from countries with low low-carbon energy vulnerability to those with high low-carbon energy vulnerability, the coefficient and significance level of the ICT service industry show an upward trend. The weakening effect of the ICT service industry on low-carbon energy vulnerability is as follows: high low-carbon energy vulnerable countries (90% quantile) > medium low-carbon energy vulnerable countries (50%, 75% quantile) > low low-carbon energy vulnerable countries (10%, 25% quantile). This result shows that the weakening effect and the marginal contribution rate of the ICT service industry can benefit more in countries with high low-carbon energy vulnerability. It means the ICT service industry has a weakening effect on low-carbon energy vulnerability. The development of the ICT service industry accelerates the research and development (R&D) efficiency of clean energy technologies through the Internet to Things (IoT) and other technologies and smart energy management platforms; reduces the constraints of low-carbon energy technologies, and at the same time, improves the efficiency of the lean energy management of enterprises, especially energy-based enterprises; improves the accuracy of energy monitoring and forecasting; and reduces the mismatch between the supply and demand of low-carbon energy.

## 5. Analysis of the Impact Mechanism

Based on the significant estimation of the moderating effect and the mediating effect model, it has been found that technological innovation and resource consumption play a moderating effect between the development of ICT services and the vulnerability of low-carbon energy. In fact, the research and development of innovative energy transition technologies can improve energy efficiency [54] and low-carbon technological innovation can enhance energy equity to a certain extent as well as reducing fuel poverty and energy vulnerability [66].

### 5.1. Research Design

The moderating effect refers to the assumption that the relationship between the two variables *X* and *Y* is a function of the moderating variable *M*, and *M* is called the moderating variable. In other words, the moderating variable *M* affects the relationship between *X* and *Y* [67]. This section will examine the moderating effects of technological innovation, energy consumption efficiency, and the impact of the ICT service industry (ln*ict_exp*) on low-carbon energy vulnerability (ln*tindex*), and set the moderating effect benchmark model as:

(5) $$tindex_{it}=\alpha +\delta \times ict\_exp_{it}+\eta \times \sum Z_{it}+\phi_{it}+\varphi_{it}+\sigma_{i}$$

In Formula (5), *Z_it_* represents control variables, including ln*agdp*, ln*p_indty*, *e_gdp*, ln*pcrd*, ln*r_older*, and ln*r_density*. $\phi_{it}$ is the time effect, $\varphi_{it}$ is an individual effect, $\sigma_{i}$ represents a random term, and η is the coefficient of the control variable. Furthermore, *r_rd2* and *re_gdp* are the moderating variables, the interaction terms *M*_1_ (ln*ict_exp* × *r_rd2*) and *M*_2_ (*ict_exp* × *re_gdp*) are added to Equation (5), and *η* represents the coefficients accordingly. We obtain:

(6) $$tindex_{it}=\alpha_{1}+\delta_{1}\times ict\_exp_{it}+\eta_{1}\times \sum Z_{it}+\theta_{1}\times M_{1}+\phi_{it}+\varphi_{it}+\sigma_{i}$$

(7) $$tindex_{it}=\alpha_{2}+\delta_{2}\times ict\_exp_{it}+\eta_{2}\times \sum Z_{it}+\theta_{2}\times M_{2}+\phi_{it}+\varphi_{it}+\sigma_{i}$$

### 5.2. The Moderating Effect of Technological Innovation

Table 6 shows the estimation results of the moderating effect model. We can see that after adding ICT service exports and technological innovation, the coefficient signs and significance levels of the control variables are basically the same, indicating that the introduction of ICT service exports and technological innovation did not change the relationship between other control variables and low-carbon energy vulnerability. At the same time, in column (11), the main effect and moderating effect are added to the OLS model. The regression coefficients of the main effect ln*ict_exp* and moderating effect ln*r_rd2* are both significantly negative at the 1% confidence level. The estimated coefficients and significances in column (12) to column (16) are basically the same, indicating that the main effect of the ICT service industry and the moderating effect of technological innovation are established, which suggests that technological innovation in OECD countries has improved, especially regarding energy intelligence, digitalization, and technological innovation capabilities. The increase in energy consumption is conducive to reducing low-carbon energy vulnerability.

Furthermore, in columns (12), (14), and (16), the estimated coefficients of ln*ict_exp* × ln*r_rd2* are all significantly negative at the 1% confidence level. The coefficient values are all greater than −0.477, indicating the interaction term has strengthened the weakening effect of the ICT service industry on low-carbon energy vulnerability and the enhanced moderating effect exerted by technological innovation. Hypothesis 2 is established. The higher the level of technological innovation, the more technological intellectual property rights a country or region has, and the better it is able to master technologies, such as renewable energy, which are related to the ICT service industry, such as chips and 5th Generation (5G), but are not easy to catch up on in a short time, and promote the application and transformation of related low-carbon energy intelligent equipment, especially the more mature IoT technology related to low-carbon energy. The more convenient the application of ICT services in low-carbon energy, the more prominent the performance of big data analysis cloud computing. Carrier applications, such as machine learning and smart energy systems, will speed up the intelligent transformation of regional energy, balance the regional energy supply and demand structure, improve the efficiency of regional low-carbon energy utilization, and reduce the low-carbon energy vulnerability of the country or region.

Attention must be paid to the effect size of the moderating effect of technological innovation, that is, the change value of R^2^. When the interaction term of ln*ict_exp* and ln*r_rd2* is added to the regression, the size of the change value of R^2^ represents the adjustment effect size. It is generally believed that R^2^ should be increased by at least 0.02 or 0.03 to consider the interaction effect to be meaningful. Otherwise, even if the significant adjustment effect is too small, it lacks practical significance. After adding technological innovation in the model of OLS (column 12) and IV-GMM (column 16), R^2^ increased by 0.065 and 0.077, respectively. This indicates that technological innovation has a significant moderating effect on low-carbon energy vulnerability.

### 5.3. Moderating Effect of Resource Consumption

In Table 7, after adding ICT service exports and resource consumption, the positive and negative coefficients of the control variables and the degree of significance are basically the same. This suggests that the introduction of ICT service exports and resource consumption do not change the relationship between the control variables and low-carbon energy vulnerability. At the same time, in column (18), the main effect and moderating effect are added to the OLS analysis. The main effect ln*ict_exp* coefficient is significantly negative at the 1% confidence level, and columns (20) and (22) are also significantly negative. The text is consistent, but the regression results of resource consumption are insignificant.

Furthermore, in columns (19), (21), and (23), the estimated coefficient of ln*ict_exp* × ln*re_gdp* is significantly positive at the 1% confidence level, indicating that the introduction of resource consumption efficiency obviously reversed the weakening effect of ICT service industry on low-carbon energy vulnerability. This can be regarded as the reversing moderating effect of resource consumption efficiency. Hypothesis 3 is established. In a region with high energy consumption efficiency, the level of low-carbon energy technology appears mainly at the stage of extensive development, especially at the level of clean energy technologies, such as renewable and nuclear energy, while the ICT of IoT smart energy, such as 5G, is low. The low conversion rate of the application of services in the field of low-carbon energy constrains the development of the ICT service industry in mitigating low-carbon energy vulnerability. At the same time, under the reality of the gradual increase in global energy demand and the Paris Agreement on carbon emissions, the higher the regional energy consumption rate, the higher the regional fossil energy demand, the weakening of the sustainability of the regional energy structure, and the majority of non-renewable fossil energy. When fossil energy supply is restricted by external factors, such as politics and weather, it will significantly increase the low-carbon energy vulnerability in the region.

After adding moderating variables, the R^2^ (effect size) of OLS and IV-GMM regression models increased by 0.035 and 0.039, respectively, indicating that the moderating effect is meaningful.

## 6. Conclusions and Policy Implications

### 6.1. Conclusions

This paper constructs a low-carbon energy vulnerability assessment system in the three dimensions of economy–society–environment, uses the entropy method to measure the low-carbon energy vulnerability level of 24 OECD countries from 2002 to 2018, and analyzes and verifies the weakening effect of the development of the ICT service industry on the low-carbon energy vulnerability. Regarding the weakening effect of the development of the ICT service industry, we discussed the moderating effects of technological innovation and resource consumption. We conclude as follows: first, the low-carbon energy vulnerability of OECD countries has shown a gradual downward trend, showing a “continuous rise–fluctuation decline–low-level volatility”. Low-carbon energy policies and implementation efforts in different countries have become the key to reducing the low-carbon energy vulnerability of OECD countries. At the same time, the types of low-carbon energy in different countries are quite different. There are fewer low low-carbon energy countries; many medium low-carbon energy vulnerabilities, with higher low-carbon energy social vulnerability; and relatively many countries with high low-carbon energy vulnerabilities and high-carbon energy economic vulnerability.

Second, the ICT service industry development coefficient is significantly negative at the 1% confidence level. Endogenous problems weaken the impact of the ICT service industry development on low-carbon energy vulnerability, indicating that the former is conducive to reducing the latter. At the same time, the ICT service industry has a weakening effect on low-carbon energy vulnerability, which can benefit more countries with high low-carbon energy vulnerability. At the same time, countries with high low-carbon energy vulnerability have a high marginal contribution rate to the ICT service industry. This is expressed as high and low-carbon energy fragile countries > medium low-carbon energy fragile countries > weak low-carbon energy fragile countries. In addition, per capita GDP and fiscal revenue and expenditure gap have a significant negative impact on the vulnerability of low-carbon energy. Industrial development level, fiscal revenue and expenditure gap, credit ratio, and population density have a significant positive impact.

Third, technological innovation and resource consumption play a moderating effect in the weakening effect of ICT’s low-carbon energy vulnerability. Among them, technological innovation plays an enhanced moderating role and the level of technological innovation is improved, especially energy intelligence, digitalization, and technological innovation. The improvement of capacity is conducive to reducing energy vulnerability; resource consumption plays a subversive moderating role: when energy consumption efficiency is high, the regional ICT industry is relatively low, and the degree of transformation and application of smart energy is low. High energy dependence is not conducive to reducing low-carbon energy vulnerability.

### 6.2. Policy Implications

Presently, the low-carbon energy transition has become most countries’ common choice. At the same time, the ICT service industry is gradually accelerating, which is helpful in accelerating the weakening effect of low-carbon energy vulnerabilities in OECD countries. Based on the research results, the following policy recommendations are proposed:

The first is to increase the low-carbon energy transition of OECD countries. Currently, the low-carbon energy vulnerability of OECD countries remains at a relatively low level, with the development of different countries being quite different. For this reason, it is necessary to advocate that OECD countries continue to adhere to green and low-carbon energy under the basic framework of the Paris Agreement and transform their development direction; formulate a more detailed roadmap regarding global temperature rise targets, capital investment, technology research and development, energy transparency, and loss and damage; and strengthen institutional guarantees, such as legislation. At the same time, for OECD countries with high low-carbon energy vulnerability, it can accelerate the proportion of renewable energy, such as hydropower, solar energy, and nuclear energy in economic energy consumption, and gradually reduce the energy intensity of unit economic development; for low-carbon energy, the vulnerability is moderate. In OECD countries, we should focus on the livelihood of low-carbon energy, especially by increasing the proportion of renewable energy per capita, and continuously improving the inclusiveness of green and low-carbon energy.

The second is to reduce the low-carbon energy vulnerability through the development of the ICT service industry and build a low-carbon energy information platform based on IoT technology to realize timely interactive communication of low-carbon energy production and sales information, especially the electric energy intelligent interactive platform for enterprises and the public. At the same time, this method uses cloud computing, big data, and machine learning. Technology such as tracking low-carbon energy consumption from time to time and self-learning, the application of demand response and sensor technology, and other automated operating systems complete the required energy output forecast, realize energy forecast automation, distribute power generation, and smart grid energy, and achieve the balance of supply and demand of low-carbon energy. Thus, energy waste is avoided, energy production and consumption efficiency are improved, and low-carbon energy vulnerability is reduced.

The third method is to accelerate technological innovation to improve energy efficiency and reduce low-carbon energy vulnerability [68]. The level of technological innovation capability directly affects the weakening effect of the ICT service industry on low-carbon energy vulnerability. On the one hand, continuous technological innovation related to the ICT service industry is required, strengthening the construction of digital infrastructure with 5G and other technologies and continuing to build an enterprise-level low-carbon energy IoT interconnection system that includes energy sales services, distributed energy services, energy conservation, and emission reduction, and demand response services, so as to increase energy demand and energy supply intelligence. At the same time, the green production and operation of ICT technologies, such as smart terminals, wireless access, fixed access, data communications, optical transmission, and smart computing, should be accelerated and the energy-saving and emission-reduction efficiency of ICT hardware facilities and green ICT solutions should be promoted. On the other hand, gradually increasing large-scale wind power generation equipment, cost-effective solar photovoltaic cell technology, fuel cell technology, biomass energy technology, hydrogen energy technology, and other renewable energy and new energy technologies, as well as carbon capture and storage technology and green cities energy-saving and energy-efficiency technologies in the fields of smart cities, chemicals, metallurgy, etc., can accelerate the transformation and application of scientific and technological achievements and continuously reduce the low-carbon energy vulnerability.

The fourth path is to reduce the efficiency of regional resource consumption continuously. Resource consumption efficiency has a subversive moderating effect on the weakening effect of low-carbon energy vulnerability in the ICT service industry. Reducing energy consumption efficiency has apparent practical value. On the one hand, there are benefits to continuing to optimize the economic structure and accelerate the transfer of energy-intensive industries, such as metals and cement, to low-energy-intensive manufacturing and service industries. At the same time, the establishment of energy service companies, green banks, and green bonds will encourage and support enterprises to promote green technological innovation, as well as the green transformation of cities; better utilize the functions and roles of ICT service carriers, such as the industrial internet, energy internet, and IoT technologies; and continuously reduce the efficiency of resource consumption. On the other hand, the optimization of the energy structure should be continued, as well as increasing the proportion of renewable energy, such as solar, wind, and hydropower in the regional energy consumption structure; reducing the proportion of fossil energy consumption, such as petroleum; and reducing carbon intensity, thereby creating a positive energy consumption environment for the ICT serving industry to reduce low-carbon energy vulnerability.

## Figures and Tables

**Table 1:** The low-carbon energy vulnerability assessment index system.

Dimension	Code	Indicator	Unit	Symbol Direction
Social	SOL1	Per capita oil consumption	Ton per capita	−
	SOL2	Per capita nature gas consumption	Ton per capita	−
	SOL3	Per capita coal consumption	Ton per capita	−
	SOL4	Per capita electricity consumption	10,000 kWH per captia	+
	SOL5	Per capita renewable energy consumption	10,000 kW per capita	+
Economic	ECO1	Oil consumption per unit GDP	Ton/USD	−
	ECO2	Nature gas consumption per unit GDP	Ton/USD	+
	ECO3	Coal consumption per unit GDP	Ton/USD	−
	ECO4	Electricity consumption per unit GDP	Ten kilowatt hours/USD	+
	ECO5	Renewable energy consumption per unit GDP	Ten kilowatt hours/USD	+
	ECO6	Proportion of the net energy import in energy consumption	%	−
	ECO7	Proportion of the flammable renewable energy and waste in total energy consumption	%	+
Environment	EVI1	Carbon emission produced by the energy consumption	Million metric tons	−
	EVI2	CO_2_ emission per unit GDP	Metric tons/USD	−

**Table 2:** Methodology and analysis of the low-carbon energy vulnerability assessment.

Methods	Characteristics	References
A modified MLBoD model	With desirable and reverse indicators construct composite indicators	Genave, Blancard, and Garabedian (2020) [2]
Principal component analysis (PCA)	PCA was performed to further reduce the dimensions of the pillars and high correlations be displayed	Gatto and Busato (2019) [3]
Entropy method	Can calculating comprehensive index system index composed of many indexes	Liu, Lan-Cui, and Wu, Gang (2014) [8]
Analytic hierarchy process (AHP)	Relative priority of each criterion with respect to each of the others is derived by a pairwise comparison using a numerical scale	Neofytou, H., and Nikas, Alexandros, and Doukas, H. (2020) [57]

**Table 3:** Descriptive statistical analysis of samples.

Variable	Mean	Std. Dev.	Min	Max
ln*tindex*	3.7401	0.2195	3.2901	4.5743
ln*ict_exp*	1.8930	0.8590	−1.5670	3.8326
ln*intser*	6.0670	2.2292	0.1271	11.5187
ln*iv_moble*	4.6184	0.3367	2.4030	5.1485
ln*re_gdp*	0.3581	0.5392	−0.9203	2.1330
ln*r_rd2*	6.8878	2.2701	2.7726	12.6541
ln*p_indty*	3.1950	0.2086	2.6161	3.6956
ln*agdp*	10.1611	0.7897	7.7325	11.5431
ln*r_older*	2.6360	0.4213	1.4270	3.1364
ln*r_density*	4.4412	1.1383	1.2380	6.2373
*e_gdp*	2.0136	4.1802	−20.3348	13.8730
ln*pcrd*	4.7390	0.4955	3.4230	5.5202

**Table 4:** The estimate result of the baseline model.

Variable	(1)	(2)	(3)	(4)
	OLS	OLS	2SLS	IV-gmm
ln*ict_exp*	−0.0845 ***	−0.0477 ***	−0.0487 **	−0.0578 ***
	(−5.65)	(−3.21)	(−2.18)	(−3.40)
ln*agdp*		−0.514 ***	−0.504 ***	−0.255 ***
		(−10.05)	(−8.09)	(−6.74)
ln*p_indty*		0.217 **	0.200 *	0.0715
		(2.09)	(1.94)	(0.71)
*e_gdp*		−0.00180	−0.00349	−0.00480 *
		(−0.62)	(−1.30)	(−1.89)
ln*pcrd*		0.0444	0.0455	0.0848 **
		(1.17)	(1.23)	(2.21)
ln*r_older*		0.0281	0.0187	−0.303 ***
		(0.18)	(0.12)	(−3.16)
ln*r_density*		0.724 ***	0.787 ***	0.524 ***
		(3.80)	(4.06)	(2.87)
_cons	3.895 ***	4.638 ***	3.743 ***	
	(119.93)	(5.16)	(3.27)	
Year	Yes	Yes	Yes	Yes
Country	Yes	Yes	Yes	Yes
N	408	408	384	384
R^2^	0.198	0.398	0.868	0.270

Note: t statistics in parentheses, * *p* < 0.1, ** *p* < 0.05, *** *p* < 0.01.

**Table 5:** The estimate result of the Sivqr model.

Variable	(5)	(6)	(7)	(8)	(9)
ln*ict_exp*	−0.175 ***	−0.166 ***	−0.150 *	−0.122	−0.0630 ***
	(−6.28)	(−3.42)	(−1.81)	(−1.06)	(−2.78)
ln*agdp*	−0.0264	−0.0378	−0.0560 *	0.0450	−0.0500
	(−0.68)	(−0.98)	(−1.83)	(0.69)	(−1.47)
ln*p_indty*	0.324 ***	0.304 ***	0.238 *	0.0844	0.293 **
	(3.49)	(2.63)	(1.67)	(0.63)	(2.51)
*e_gdp*	0.0119 ***	0.0139 ***	0.0149 **	0.0226 **	0.0149 **
	(3.31)	(3.72)	(2.43)	(2.12)	(2.23)
ln*pcrd*	−0.0377	−0.0231	−0.0330	−0.188 ***	−0.139 ***
	(−0.73)	(−0.41)	(−1.01)	(−3.13)	(−3.02)
ln*r_older*	0.213 ***	0.0415	−0.0503	−0.259 **	−0.215 ***
	(4.48)	(0.43)	(−0.65)	(−2.52)	(−5.27)
ln*r_density*	0.00247	−0.00146	−0.0210	−0.0320 ***	−0.00851
	(0.31)	(−0.12)	(−1.41)	(−3.48)	(−0.81)
_cons	2.665 ***	3.322 ***	4.171 ***	5.035 ***	4.874 ***
	(5.25)	(5.04)	(5.06)	(5.41)	(9.79)
N	408	408	408	408	384

Note: t statistics in parentheses, * *p* < 0.1, ** *p* < 0.05, *** *p* < 0.01.

**Table 6:** Estimated results of the moderating effect of technological innovation.

Variable	(10)	(11)	(12)	(13)	(14)	(15)	(16)
	OLS	OLS	OLS	2SLS	2SLS	IV-GMM	IV-GMM
ln*ict_exp*		−0.0542 ***	−0.0447 ***	−0.0548 **	−0.0462 **	−0.0610 ***	−0.0457 ***
		(−3.60)	(−3.12)	(−2.34)	(−2.42)	(−3.41)	(−2.66)
ln*r_rd2*		−0.0206 **	−0.0255 ***	−0.0174 *	−0.0205 **	−0.00739	−0.0137
		(−2.22)	(−2.90)	(−1.86)	(−2.26)	(−0.69)	(−1.36)
ln*ict_exp* × ln*r_rd2*			−0.0447 ***		−0.0435 ***		−0.0473 ***
			(−6.60)		(−4.81)		(−6.46)
ln*agdp*	−0.560 ***	−0.553 ***	−0.455 ***	−0.533 ***	−0.440 ***	−0.262 ***	−0.212 ***
	(−11.26)	(−10.28)	(−8.58)	(−7.99)	(−7.28)	(−6.73)	(−5.66)
ln*p_indty*	0.239 **	0.212 **	0.263 ***	0.202 *	0.222 **	0.0743	0.0791
	(2.28)	(2.05)	(2.68)	(1.95)	(2.24)	(0.73)	(0.82)
*e_gdp*	−0.00240	−0.00267	0.000647	−0.00442	−0.000817	−0.00499 *	−0.00285
	(−0.82)	(−0.91)	(0.23)	(−1.59)	(−0.30)	(−1.95)	(−1.17)
ln*pcrd*	0.0520	0.0301	−0.0591	0.0342	−0.0566 *	0.0801 **	−0.0181
	(1.35)	(0.78)	(−1.53)	(0.90)	(−1.65)	(2.05)	(−0.45)
ln*r_older*	0.142	0.0698	−0.238	0.0410	−0.264 *	−0.314 ***	−0.460 ***
	(0.92)	(0.44)	(−1.53)	(0.26)	(−1.78)	(−3.23)	(−4.87)
ln*r_density*	0.991 ***	0.875 ***	0.829 ***	0.908 ***	0.829 ***	0.559 ***	0.649 ***
	(5.71)	(4.35)	(4.35)	(4.58)	(4.57)	(2.97)	(3.64)
_cons	3.437 ***	4.489 ***	4.783 ***	3.487 ***	4.194 ***		
	(4.15)	(5.00)	(5.63)	(3.18)	(4.37)		
N	408	408	408	384	384	384	384
R^2^	0.381	0.406	0.471	0.869	0.882	0.271	0.348

Note: In parentheses are the t statistics, * *p* < 0.1, ** *p* < 0.05, *** *p* < 0.01.

**Table 7:** Estimated results of resource consumption effect.

Variable	(17)	(18)	(19)	(20)	(21)	(22)	(23)
	OLS	OLS	OLS	2SLS	2SLS	IV-GMM	IV-GMM
ln*ict_exp*		−0.0465 ***	−0.0183	−0.0497 **	−0.0168	−0.0641 ***	−0.0290
		(−3.06)	(−1.16)	(−2.11)	(−0.82)	(−3.61)	(−1.47)
ln*re_gdp*		0.0422	0.0285	−0.0279	−0.0358	−0.113	−0.143
		(0.42)	(0.29)	(−0.34)	(−0.46)	(−1.23)	(−1.59)
ln*ict_exp* × ln*re_gdp*			0.127 ***		0.125 ***		0.127 ***
			(4.77)		(3.44)		(4.29)
ln*agdp*	−0.560 ***	−0.523 ***	−0.508 ***	−0.498 ***	−0.499 ***	−0.242 ***	−0.246 ***
	(−11.26)	(−9.41)	(−9.40)	(−7.89)	(−7.82)	(−6.15)	(−6.41)
ln*p_indty*	0.239 **	0.199 *	0.197 *	0.212 *	0.216 *	0.148	0.146
	(2.28)	(1.76)	(1.80)	(1.91)	(1.95)	(1.25)	(1.27)
*e_gdp*	−0.00240	−0.00197	−0.000728	−0.00337	−0.00229	−0.00402	−0.00362
	(−0.82)	(−0.67)	(−0.25)	(−1.26)	(−0.83)	(−1.53)	(−1.42)
ln*pcrd*	0.0520	0.0436	−0.000203	0.0461	0.00853	0.0809 **	0.0443
	(1.35)	(1.14)	(−0.01)	(1.24)	(0.25)	(2.10)	(1.16)
ln*r_older*	0.142	0.0172	−0.165	0.0260	−0.127	−0.331 ***	−0.412 ***
	(0.92)	(0.11)	(−1.03)	(0.16)	(−0.81)	(−3.36)	(−4.22)
ln*r_density*	0.991 ***	0.710 ***	0.589 ***	0.795 ***	0.700 ***	0.520 ***	0.486 ***
	(5.71)	(3.67)	(3.10)	(4.04)	(3.69)	(2.85)	(2.74)
_cons	3.437 ***	4.532 ***	5.633 ***	3.799 ***	4.870 ***		
	(4.15)	(4.85)	(6.01)	(3.33)	(4.59)		
N	408	408	408	384	384	384	384
R^2^	0.381	0.399	0.434	0.868	0.876	0.273	0.312

Note: T statistics are in parentheses, * *p* < 0.1, ** *p* < 0.05, *** *p* < 0.01.

## Data Availability

Data is available on request.

## Nomenclature

OECD	Organization for Economic Co-operation and Development
ICT	Information Communication Technology
COVID-19	Coronavirus Disease 2019
CCUS	Carbon Capture, Utilization, and Storage
IEA	International Energy Agency
EU	European Union
UN	United Nations
US	United States
USD	United States Dollar
GDP	Gross Domestic Product
GEVI	Global Energy Vulnerability Index
EPS	Economy Prediction System
CAFE	Corporate Average Fuel Economy
Sivqr	Smoothed IV quantile regression
IMF	International Monetary Fund
IoT	Internet of Things
R&D	Research and Development
OLS	Orthogonal Least Square
2SLS	Two Stage Least Square
IV-GMM	Independent Variable Gaussian Mixed Model
5G	5th Generation
